# Mediating effects of grit and perceived social support between resilience and post-traumatic growth in the emergency room nurses

**DOI:** 10.3389/fpsyg.2025.1535603

**Published:** 2025-09-15

**Authors:** Eun-Hee Min, Hyung-Ran Park

**Affiliations:** Department of Nursing Science, Research Institute of Nursing Science, College of Nursing, Chungbuk National University, Cheongju, Republic of Korea

**Keywords:** resilience, post-traumatic growth, grit, social support, emergency room

## Abstract

Emergency room (ER) nurses are frequently exposed to traumatic events, leading to mental fatigue and reduced quality of life. However, to overcome such events, some nurses exhibit positive psychological transformation and behavioural change, known as post-traumatic growth (PTG). This cross-sectional descriptive study examined the mediating effects of grit and perceived social support on the relationship between resilience and PTG among ER nurses. Data were collected from 182 ER nurses across seven general hospitals in Chungcheong Province, South Korea. SPSS version 26.0 and the PROCESS macro program (MODEL 6) were used for data analysis. The results indicated that resilience significantly affected ER nurses' PTG. Furthermore, grit and perceived social support mediated this relationship. The findings emphasise the importance of enhancing resilience, grit, and perceived social support to promote ER nurses' PTG.

## 1 Introduction

Among nurses, those in the emergency room (ER) are more directly exposed to traumatic events such as death and workplace violence, which they repeatedly encounter in a complex and intense work environment while caring for severely injured patients (de Snoo et al., 2022; Jung and Park, 2021). Repeated exposure to traumatic events can have a detrimental effect on ER nurses' work, which can manifest in mental fatigue, avoidance, and workplace attrition; additionally, such exposure can decrease their overall quality of life, as evidenced by symptoms such as difficulty sleeping, fear, anger, and worry (Rodney et al., 2022). However, not all nurses who experience traumatic events react negatively. Some nurses who experience a traumatic event go through an adjustment period in which they actively cope with and overcome the trauma (Jiang et al., 2024; Jung and Park, 2021; Liu et al., 2024). Post-traumatic growth (PTG) refers to the positive psychological transformation and behavioural change that individuals experience after exposure to a traumatic event (Tedeschi and Calhoun, 1996). This concept encompasses five domains: personal strength, interpersonal relationships, appreciation of life, new possibilities, and spiritual and existential changes (Tedeschi and Calhoun, 1996). Identifying the factors associated with ER nurses' PTG is crucial to fostering growth following exposure to traumatic events.

Resilience has been recognised as a significant PTG-influencing factor among nurses who experienced trauma (Jung and Park, 2021; Tang et al., 2024). In a study of ER nurses (Jung and Park, 2021) and in several previous studies (Han et al., 2023; O'Donovan and Burke, 2022), resilience was found to be positively associated with PTG, and it fostered growth by enabling coping with trauma-induced stress (Jiang et al., 2024; Liu et al., 2024). Resilience refers to an individual's ability to recover when faced with challenging events such as adversity (Connor and Davidson, 2003). It facilitates recovery after traumatic events by coping well and developing adaptive strategies (Biggs et al., 2024). Notably, resilience does not simply assist one in returning to the state before the traumatic event but rather incorporates growth changes after learning to adapt through the experienced adversity (Biggs et al., 2024). Therefore, it is important to determine resilience's effect on PTG. Previous studies suggested that resilience influences PTG through various variables, including nurses' meaning in life (Tang et al., 2022) and various coping mechanisms such as social support, avoidance, positive attitude, approach coping, and religious coping in cancer patients (Gori et al., 2020).

Grit, a positive coping strategy, refers to individuals' ability to maintain effort and interest when facing adversity and to persistently use strategies necessary to progress towards achieving long-term goals in a given situation (Duckworth et al., 2007). Resilience and grit are controversially used terms in nursing (Biggs et al., 2024), and grit is considered a distinct construct from resilience; however, several studies reported a correlation between the two (Al-Zain and Abdulsalam, 2022; Hamdan et al., 2023; Lee et al., 2023; Musso et al., 2019). When faced with traumatic events, gritty nurses grow by persevering over time (Biggs et al., 2024). A previous study indicated that grit was a significant predictor of lower post-traumatic stress disorder (PTSD) symptoms among emergency medical service personnel (Musso et al., 2019). In studies of healthcare students, higher levels of grit were found to be associated with lower reported stress (Al-Zain and Abdulsalam, 2022; Kim and Lee, 2022). Cultivating grit in ER nurses who frequently experience traumatic events may enable them to better cope with traumatic events and even thrive (Tyer-Viola, 2019). Therefore, examining the effect of grit-mediated resilience on PTG is meaningful for ER nurses.

After experiencing a traumatic event, individuals grow post-traumatically by recognising their vulnerability, expressing their feelings to those who accept them, and utilising social support that was previously ignored (Tedeschi and Calhoun, 1996). Perceived social support occurs when an individual experiences subjective feelings of being understood, respected, and supported by others (O'Donovan and Burke, 2022). Social support has two sources: extrinsic resources, such as family and friends, and intrinsic resources, such as co-workers and leaders (O'Donovan and Burke, 2022). Perceived social support was a significant factor that influenced PTG among nurses who experienced trauma during the COVID-19 pandemic (Mo et al., 2022). Trauma nurses with higher levels of perceived social support reported lower levels of PTSD while caring for trauma patients (Kim and Yeo, 2020). Studies involving natural disasters and traumatic events found that perceived social support was highly correlated with resilience (Cheng et al., 2024; Xi et al., 2020; Yang and Bae, 2022) and was suggested as a mediator of coping with post-trauma stress (Xi et al., 2020). Additionally, perceived social support can promote PTG when individuals share their vulnerabilities and seek help from a support system that they perceive as beneficial after experiencing a traumatic event (Cheng et al., 2024; Mo et al., 2022; Tedeschi and Calhoun, 1996; Xi et al., 2020; Yang and Bae, 2022), indicating the need for identifying the effects of resilience on PTG mediated by perceived social support. Additionally, grit, when combined with social support, may help reduce academic stress in nursing students and foster positive emotions among nurses (Kim and Lee, 2022; Yang and Wu, 2021). In conclusion, limited research has examined the mediating mechanisms through which resilience impacts PTG among ER nurses. Understanding this mechanism could inform interventions aimed at strengthening ER nurses' capacities and enhancing their ability to proficiently manage trauma and emergency situations (Liu et al., 2024). Therefore, it is crucial to determine the chain mediating effects of resilience on PTG through grit and perceived social support among ER nurses.

### 1.1 Purpose

This study examined the mediating effects of grit and perceived social support on the relationship between resilience and PTG among ER nurses. Particularly, this study aimed to determine the extent of PTG according to participants' general characteristics, analyse the correlations between resilience, grit, and perceived social support, and examine the chain mediating effects of grit and perceived social support on the relationship between resilience and PTG.

## 2 Materials and methods

### 2.1 Research design

A cross-sectional survey design was used to explore the relationship between resilience and PTG, as well as the mediating effects of grit and perceived social support among ER nurses. Specifically, we hypothesised that ER nurses' grit and perceived social support mediate the effect of resilience on PTG, such that higher levels of grit and perceived social support would strengthen the association between resilience and PTG among ER nurses.

### 2.2 Participants

The study sample comprised 182 nurses, selected from seven general hospitals in Chungcheong Province, who had at least 6 months of experience in the ER and had faced traumatic events. Since almost all ER nurses report experiencing a traumatic event (Qian et al., 2023), a selection criterion of 6 months was set to consider the period during which such an event could occur. Using G^*^Power 3.1.9.2, the sample size was calculated with a significance level of 0.05, a medium effect size of 0.15, a power of 0.95, and three predictors and 13 general characteristics for multiple regression. The results indicated that a sample of 175 was required. After accounting for a 10% dropout rate, a total of 193 participants were surveyed using convenience sampling, and data from 182 participants were used in the final analysis, after excluding 11 participants with incomplete data.

### 2.3 Measures

This study employed structured questionnaires, with permission from the respective original authors. Participants' sociodemographic and work-related characteristics were measured using a self-reported questionnaire comprising 13 items adapted from previous studies (O'Donovan and Burke, 2022; Tang et al., 2024).

#### 2.3.1 Resilience

Resilience was measured using the purchased Korean version of the Connor-Davidson Resilience Scale (Connor and Davidson, 2003), which was translated and validated by (Baek et al. 2010) for use with hospital nurses, university students, and firefighters. The scale comprises five domains: hardiness, perseverance, optimism, support, and spirituality, with 25 questions rated on a 5-point Likert scale ranging from 0 (not at all) to 4 (very much so), with total scores ranging from 0 to 100. Higher scores indicate higher resilience. The Cronbach's α was 0.93 in both Baek et al.'s study (Baek et al., 2010) and in the present study.

#### 2.3.2 Grit

Based on the grit tool developed by (Duckworth et al. 2007), we assessed grit using the Clinical Nurses Grit Scale (CN-GRIT) developed and validated by (Park et al. 2020) to reflect the Korean culture and clinical nursing environment. The scale comprises 14 items in three domains: persistence to achieve long-term goals, passion to become a nursing professional, and patient-oriented intrinsic motivation. Each item is rated on a 4-point Likert scale ranging from 1 (not always) to 4 (always), with total scores ranging from 14 to 56. Higher scores indicate higher levels of grit. The Cronbach's α was 0.78 in the original study by (Duckworth et al. 2007), 0.91 in the study by (Park et al. 2020), and 0.91 in this study.

#### 2.3.3 Perceived social support

Perceived social support was measured using the Perceived Social Support Scale developed by (Park 1985) and modified and supplemented by (Yu 2012). The scale comprises 25 items in four domains: emotional, appraisal, informational, and material support. Each item is rated on a 5-point Likert scale ranging from 1 (not at all) to 5 (very much), with total scores ranging from 25 to 125. Higher scores indicate greater perceived social support. Cronbach's α was 0.94 in Park's (1985) study, 0.97 in Yu's (2012) study, and 0.96 in (Yeo and Park 2020) study on psychiatric nurses. Cronbach's α was 0.97 in this study.

#### 2.3.4 Post-traumatic growth (PTG)

PTG was measured using the Post-Traumatic Growth Inventory, which was developed by (Tedeschi and Calhoun 1996) and validated and translated to Korean by (Song et al. 2009). This tool comprises 16 items in four domains: changes in self-perception, changes in interpersonal relationships, discovery of new possibilities, and spiritual growth. Each item is rated on a 6-point Likert scale ranging from 0 (not at all) to 5 (very much), with total scores ranging from 0 to 80. Higher scores indicate more positive post-traumatic changes. Cronbach's α was 0.90 in the study by (Tedeschi and Calhoun 1996), 0.91 in Song et al.'s (2009) study, and 0.92 in the present study.

### 2.4 Data collection

This study was approved by the Institutional Review Board of Chungbuk National University, and the data were collected from July 9 to August 8, 2024. The participants were ER nurses from seven general hospitals located in Chungcheong Province who voluntarily participated after being informed of the study's purpose and methods. Additionally, the documented description of the study explicitly stated that its purpose was to identify factors affecting the growth of ER nurses in response to traumatic events, allowing nurses to participate based on their experiences with traumatic events. With the cooperation of each respective hospital and nursing department, paper-based self-reported questionnaires were distributed and later collected in individual sealed envelopes. The completed questionnaires were collected by the researcher and assigned arbitrary identification numbers to ensure participant anonymity. The participants were informed of the expected benefits and risks of participating in the study, that they could withdraw from the study at any time, and that there would be no penalty for withdrawing. Additionally, they were informed that the collected data would be encoded to ensure anonymity, would not be used for any purpose other than the study, and would be kept for 3 years after the study ended for future inspection before being destroyed.

### 2.5 Data analysis

The collected data were analysed using the SPSS Statistics 26.0 program (IBM Corp., Armonk, NY, USA) and the PROCESS macro program (https://www.processmacro.org/download.html). Participants' sociodemographic and work-related characteristics, resilience, grit, perceived social support, and PTG were analysed using descriptive statistics. Differences in PTG based on the participants' sociodemographic and work-related characteristics were evaluated using independent *t*-tests and one-way ANOVA. Correlations between the variables were analysed using Pearson's correlation coefficient. The effect of resilience on PTG, mediated by grit and perceived social support, was assessed for multicollinearity, and the direct and mediated effects were analysed by applying Hayes' (2017) PROCESS macro Model 6 (two mediators). The mediating effects were analysed while controlling for marital status, religion, satisfaction with working in the ER, intention to change departments, and monthly income, as these showed significant associations with PTG among the sociodemographic and work-related characteristics. Indirect effects were examined using 10,000 bias-adjusted bootstrapping with 95% confidence intervals.

## 3 Results

### 3.1 Differences in post-traumatic growth according to sociodemographic and work-related characteristics

A total of 182 ER nurses were included in this study, with a mean age of 29.72 ± 5.84 years. Regarding sociodemographic characteristics, 45 (24.7%) participants were married, and 137 (75.3%) participants were single, with married participants reporting significantly higher PTG scores compared to single participants [t (180) = −2.74, *p* = 0.007]. Additionally, 46 (25.3%) participants were religious and 136 (74.7%) were not; religious participants reported higher PTG scores compared to non-religious participants [t (180) = 3.56, *p* < 0.001]. Regarding work-related characteristics, 121 (66.5%) participants were satisfied with their current department, and 61 (33.5%) were moderately satisfied; satisfied participants had significantly higher PTG scores compared to moderately satisfied participants [t (180) = −3.60, *p* < 0.001]. In addition, 41 (22.5%) participants had intentions of changing departments and 141 (77.5%) did not [t (180) = −2.20, *p* = 0.029]. Overall, 85 (46.7%) participants had a monthly income of < 3 million won and 97 (53.3%) had a monthly income of ≥3 million won. Participants whose monthly income was more than 3 million won had higher PTG scores compared to those with a monthly income < 3 million won [t (180) = −2.75, *p* = 0.007; Table 1].

**Table 1:** Differences in post-traumatic growth according to sociodemographic and work-related characteristics (*N* = 182).

**Variables**	**Categories**	***n* (%)**	**Post-traumatic growth**	
			**M** ±**SD**	***t*** **or** ***F*** **(*****p*****)**
Sex	Female	136 (74.7)	3.13 ± 0.73	−0.75 (0.452)
	Male	46 (25.3)	3.22 ± 0.60	
Age (year)	< 30	107 (58.8)	3.11 ± 0.68	0.69 (0.562)
	30– < 40	64 (35.2)	3.19 ± 0.70	
	≤ 40	11 (6.0)	3.34 ± 0.99	
Marital status	Single	137 (75.3)	3.07 ± 0.71	−2.74 (0.007)
	Married	45 (24.7)	3.40 ± 0.63	
Religion	Follow	46 (25.3)	3.46 ± 0.76	3.56 (< 0.001)
	Do not follow	136 (74.7)	3.05 ± 0.65	
Education level	College	12 (6.6)	3.22 ± 0.64	0.42 (0.658)
	University	146 (80.2)	3.13 ± 0.65	
	Master's or above	24 (13.2)	3.26 ± 1.00	
Position	Staff nurse	167 (91.8)	3.14 ± 0.69	−0.83 (0.407)
	Charge nurse	15 (8.2)	3.30 ± 0.85	
Total clinical career (year)	< 5	88 (48.4)	3.16 ± 0.63	2.12 (0.123)
	5– < 10	61 (33.5)	3.03 ± 0.74	
	≥10	33 (18.1)	3.34 ± 0.79	
Career in ER (year)	< 5	116 (63.7)	3.14 ± 0.66	0.48 (0.621)
	5– < 10	45 (24.7)	3.11 ± 0.75	
	≥10	21 (11.6)	3.29 ± 0.85	
Types of ER	Regional emergency medical centres	66 (36.3)	3.14 ± 0.57	0.01 (0.995)
	Local emergency medical centres	100 (54.9)	3.16 ± 0.77	
	Local emergency medical institutions	16 (8.8)	3.15 ± 0.79	
Requested to work in the ER	Requested	146 (80.2)	3.17 ± 0.68	0.71 (0.478)
	Not requested	36 (19.8)	3.08 ± 0.80	
Satisfaction with working in the ER	Satisfied	121 (66.5)	3.28 ± 0.70	−3.60 (< 0.001)
	Moderately satisfied	61 (33.5)	2.89 ± 0.64	
Intention to change departments	No	141(77.5)	3.21 ± 0.67	−2.20 (0.029)
	Yes	41(22.5)	2.94 ± 0.78	
Monthly income (10,000 won)	< 300	85(46.7)	3.00 ± 0.72	−2.75 (0.007)
	≥300	97(53.3)	3.28 ± 0.67	

ER, emergency room; M, mean; SD, standard deviation.

### 3.2 Scores and correlations among resilience, grit, perceived social support, and post-traumatic growth

Participants' item mean resilience was 2.57 ± 0.51 (mean of total score = 64.24 ± 12.62), grit was 3.03 ± 0.41 (42.41 ± 5.73), perceived social support was 3.78 ± 0.61 (94.55 ± 15.20), and PTG was 3.15 ± 0.70 (50.42 ± 11.25). Participants' PTG was statistically significant and had moderately positive correlations with resilience (*r* = 0.57, *p* < 0.001), grit (*r* = 0.57, *p* < 0.001), and perceived social support (*r* = 0.54, *p* < 0.001; Table 2).

**Table 2:** Scores and correlations among variables (*N* = 182).

**Variables**	**Number of items**	**Item mean**		**RES**	**Grit**	**PSS**	**PTG**
		**M** ±**SD**	**Range**	**r**			
RES	25	2.57 ± 0.51	0.68–4.00	1			
Grit	14	3.03 ± 0.41	1.50–3.93	0.56^*^	1		
PSS	25	3.78 ± 0.61	1.96–5.00	0.55^*^	0.48^*^	1	
PTG	16	3.15 ± 0.70	1.06–5.00	0.57^*^	0.57^*^	0.54^*^	1

RES, resilience; PSS, perceived social support; PTG, post-traumatic growth; M, mean; SD, standard deviation; ^*^p < 0.001.

### 3.3 Effect of resilience on post-traumatic growth through the mediation of grit and perceived social support

Before testing the mediating effect of the variables, this study's assumptions were tested using multiple regression analysis, and the Durbin–Watson test value was 2.219, which was within an acceptable range (1.5–2.5), indicating no autocorrelation between each independent variable. The tolerance of each variable in the regression was between 0.48 and 0.96, which was >0.1, and the variance inflation factor was between 1.04 and 2.09, which was < 10, indicating no multicollinearity problem. To test the mediation effect of grit and perceived social support on the relationship between resilience and PTG in ER nurses, a serial binary mediation analysis was conducted using Model 6 of the PROCESS macro proposed by (Hayes 2017). The analysis controlled for marital status, religion, satisfaction with working in the ER, intention to change department, and monthly income, as these variables were expected to influence PTG among the sociodemographic and work-related characteristics (Table 3). Resilience had a direct effect on grit (β = 0.50, *p* < 0.001) and perceived social support (β = 0.41, *p* < 0.001), while grit had a direct effect on perceived social support (β = 0.31, *p* < 0.001) among ER nurses. In addition, resilience (β = 0.22, *p* = 0.008), grit (β = 0.29, *p* < 0.001), and perceived social support (β = 0.24, *p* = 0.006) had a significant direct effect on PTG. Regarding the relationship between resilience and PTG, the total effect of grit and perceived social support (β = 0.50, *p* < 0.001) was greater than the direct effect of resilience on PTG (β = 0.22, *p* = 0.008), confirming a mediating effect.

**Table 3:** Mediation effect of grit and perceived social support between resilience and post-traumatic growth.

**Variables**	**Categories**	**B**	**SE**	**β**	***t***	***p***	**95% CI**	
							**Boot LLCI**	**Boot LLCI**
Total effect		0.66	0.09	0.50	7.09	< 0.001		
Direct effect	X → M1	0.40	0.06	0.50	6.94	< 0.001		
	X → M2	0.46	0.08	0.41	5.78	< 0.001		
	M1 → M2	0.43	0.10	0.31	4.38	< 0.001		
	X → Y	0.29	0.11	0.22	2.70	0.008		
	M1 → Y	0.48	0.13	0.29	3.76	< 0.001		
	M2 → Y	0.28	0.10	0.24	2.79	0.006		
Indirect effect	X → M1 → Y	0.19	0.06^†^				0.08	0.30
	X → M2 → Y	0.13	0.06^†^				0.03	0.26
	X → M1 → M2 → Y	0.05	0.03^†^				0.01	0.12
	Total	0.37	0.09^†^				0.21	0.55

X, resilience; M1, grit; M2, perceived social support; Y, post-traumatic growth; SE, standard error; CI, confidence interval; LLCI, lower limit CI; ULCI, upper limit CI.

^†^Boot SE.

**Figure 1:**
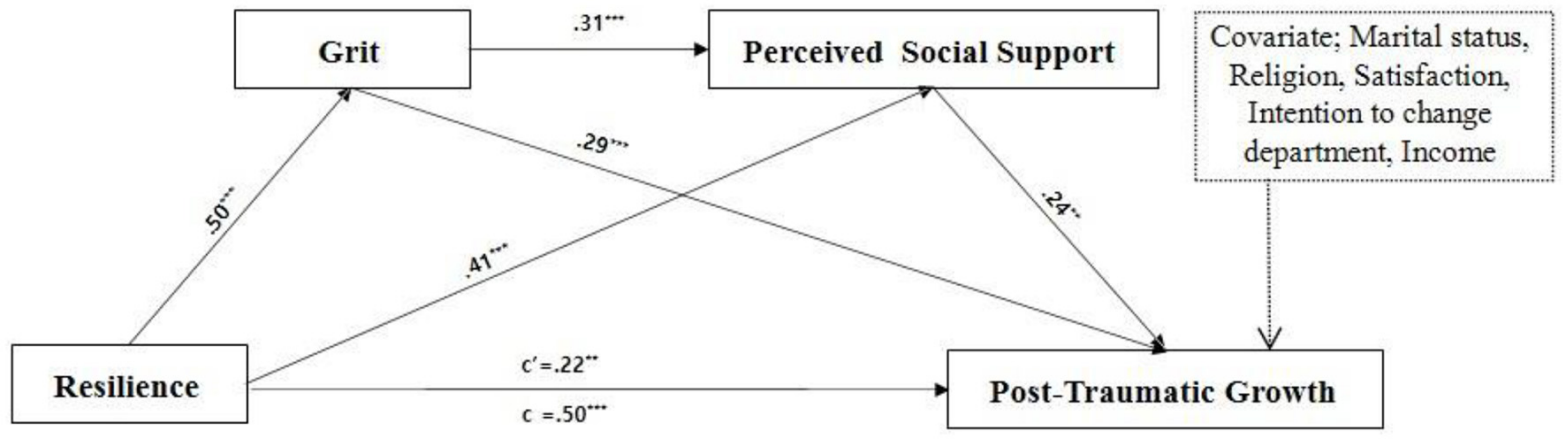
Mediating effect model of grit and perceived social support between resilience and post-traumatic growth using the PROCESS macro. *p < 0.05, **p < 0.01, ***p < 0.001. c', direct effect; c, total effect.

To determine the significance of the mediating effect of grit and perceived social support (indirect effect) on the relationship between resilience and PTG, a bootstrap was performed with 10,000 iterations. The simple mediation effect of grit on the relationship between resilience and PTG among ER nurses was statistically significant at 0.19 (0.08–0.30) for X → M1 → Y, with the upper and lower bounds of the 95% bootstrap confidence interval not including zero. Furthermore, the simple mediation effect of perceived social support on the relationship between resilience and PTG among ER nurses was statistically significant at 0.13 (0.03–0.26) for X → M2 → Y, with the upper and lower bounds of the 95% bootstrap confidence interval not including zero. The chain mediating effect of grit and perceived social support on the relationship between resilience and PTG in ER nurses was statistically significant at 0.05 (0.01–0.12) for X → M1 → M2 → Y, with the upper and lower bounds of the 95% bootstrap confidence interval not including zero (Figure 1).

## 4 Discussion

To the best of our knowledge, this study is the first to examine the mediating effect of grit and perceived social support on the relationship between resilience and PTG among ER nurses in South Korea. The results indicated that resilience has a direct effect on PTG and an indirect effect through the mediation of grit and perceived social support. This result was consistent with previous studies on factors influencing PTG (O'Donovan and Burke, 2022; Tang et al., 2024), where resilience was found to be influenced by personal factors. Resilience has been suggested to have a direct effect on PTSD in a study of nurses during the COVID-19 pandemic (Han et al., 2023). In a study of ER nurses, resilience was the most influential predictor, accounting for 31.9% to 47% of the total variance (Jung and Park, 2021), suggesting that resilience can enhance nursing performance as nurses recover and grow after experiencing a traumatic event. Resilience, which has been shown to be a major contributor to PTG in a study of nursing students, is characterised by interpersonal or intrapersonal factors that precede psychological responses to traumatic events; thus, strengthening resilience may directly improve PTG (Kim et al., 2023). Improving brain fitness, finding meaning and purpose in life, and developing resilience behaviours, which have been shown to be significantly associated with PTG in medical students (Luo et al., 2022). A study of frontline nurses during the COVID-19 pandemic in China found that resilience was positively associated with PTG, as it helped nurses adapt effectively to emergency situations (Zeng et al., 2024). According to resilience theory, nurses with higher resilience have a stronger sense of control and are more capable of problem-solving, which supports their ability to overcome crises and fosters personal growth (Khatooni et al., 2023). Moreover, resilience is often reinforced by relying on individual inner abilities, such as grit (Zeng et al., 2024), suggesting that enhancing PTG among ER nurses—who directly experience various traumatic events—is essential.

In this study, the simple mediation of grit in the relationship between resilience and PTG was found to be effective after controlling for general characteristics. Higher resilience was associated with increased PTG, which was mediated by higher levels of grit. These findings are consistent with previous studies showing that higher resilience was associated with higher levels of grit in general hospital (Lee et al., 2023) and ER nurses (Musso et al., 2019). Moreover, it is similar to how grit mediates the direction of meaning in life across a range of stressful events (Lee and Lee, 2023; Liu et al., 2022; Tyer-Viola, 2019; Yu et al., 2024). Brain imaging studies of trauma and PTG in post-COVID-19 college students suggested that functional connectivity density in the right dorsolateral prefrontal cortex, which plays a central role in self-regulation and reward-motivation processes, mediates PTG and grit (Wang et al., 2023). (Musso et al. 2019) defined resilient individuals as those who persist in the face of adversity and continue to pursue their passions, so their resilience may lead to greater levels of grit over time. Thus, ER nurses who are more resilient in dealing with traumatic events are more likely to face challenging situations with a positive perspective, set goals for problem-solving, and persist in their efforts to achieve them. Consequently, developing personal traits that allow them to persevere in stressful situations such as traumatic events, along with resilience, can assist ER nurses in thriving in the workplace.

This study found that perceived social support had a significant mediating effect on the relationship between resilience and PTG among ER nurses. This is consistent with previous studies that identified perceived social support as a key influencer of PTG (Musso et al., 2019; Tang et al., 2024) and showed that psychological support from co-workers may assist nurses in recovering from traumatic experiences (Musso et al., 2019). Furthermore, social support from co-workers may be a preventive intervention to reduce PTSD in nurses who experienced trauma (Kim and Yeo, 2020). According to the conceptual foundation of PTG, perceived social support facilitates the transformation of traumatic events into personal growth by promoting emotional wellbeing, thereby enabling ER nurses to feel more supported and experience TG (Tedeschi and Calhoun, 2004).

Additionally, perceived social support, along with grit, was found to have a chain mediating effect on the relationship between resilience and PTG. These results were similar to a previous study's findings, indicating that resilience-mediated social support reduces PTSD symptoms in those who experienced trauma caused by a natural disaster (Xi et al., 2020). With increased perceived social support, resilient nurses can avoid experiencing negative emotions and stress after a traumatic event, which may lead to PTG (Cheng et al., 2024). This study found a chain mediation effect of grit and perceived social support, which is consistent with previous studies that found grit to be a factor influencing perceived social support (Kim and Lee, 2022; Yang and Wu, 2021). High levels of grit positively impact meaning in life by enhancing nurses' perceived social support (Yang and Wu, 2021) and reducing academic burnout in nursing students (Kim and Lee, 2022), which suggests that as nurses persist in setting and achieving goals to solve problems, enhanced feelings of support from others may facilitate the processes that lead to PTG.

Finally, developing grit, along with resilience in stressful situations, such as traumatic events, and providing social support can be important factors in promoting PTG among ER nurses. Grit can be cultivated and strengthened, even in the presence of mental distress, through interventions that focus on enhancing individual abilities and encouraging the pursuit of activities of interest (Sigmundsson and Hauge, 2023). Therefore, ER units should develop programs to increase nurses' levels of grit and provide sufficient social support to promote their PTG.

This study has some limitations. First, its cross-sectional design limits the ability to assess changes in variables over time. A longitudinal approach is needed to identify the variations and causal relationships. Second, the study sample consisted solely of Korean participants, reflecting the situation in South Korean ERs. As a result, the findings have limited generalisability and should be validated in different cultural, demographic, or clinical settings. Finally, data were collected through self-reported questionnaires, which may be subject to response bias. Therefore, further studies should employ objective methods to obtain more representative and reliable data.

## 5 Conclusions

This study examined the mediating effects of grit and perceived social support on the relationship between resilience and PTG in ER nurses and explored ways in which PTG can be promoted in ER nurses. The results indicated that resilience influenced PTG in ER nurses, and during this process, grit and perceived social support had a chain mediation effect on the relationship between resilience and PTG. The findings suggested that programs that bolster resilience and grit after traumatic experiences should be developed to enhance PTG in ER nurses, and both peer and supervisory support should be provided.

## Data Availability

The raw data supporting the conclusions of this article will be made available by the authors, without undue reservation.
